# America COMPETES at 5 years: An Analysis of Research-Intensive Universities’ RCR Training Plans

**DOI:** 10.1007/s11948-017-9883-5

**Published:** 2017-03-15

**Authors:** Trisha Phillips, Franchesca Nestor, Gillian Beach, Elizabeth Heitman

**Affiliations:** 1 West Virginia University, Morgantown, WV USA; 2 University of Texas Southwestern Medical Center, Dallas, TX USA

**Keywords:** RCR, Ethics education, Research integrity policy, Research integrity

## Abstract

This project evaluates the impact of the National Science Foundation's (NSF) policy to promote education in the responsible conduct of research (RCR). To determine whether this policy resulted in meaningful RCR educational experiences, our study examined the instructional plans developed by individual universities in response to the mandate. Using a sample of 108 U.S. institutions classified as Carnegie “very high research activity”, we analyzed all publicly available NSF RCR training plans in light of the consensus best practices in RCR education that were known at the time the policy was implemented. We found that fewer than half of universities developed plans that incorporated at least some of the best practices. More specifically, only 31% of universities had content and requirements that differed by career stage, only 1% of universities had content and requirements that differed by discipline; and only 18% of universities required some face-to-face engagement from all classes of trainees. Indeed, some schools simply provided hand-outs to their undergraduate students. Most universities (82%) had plans that could be satisfied with online programs such as the Collaborative Institutional Training Initiative's RCR modules. The NSF policy requires universities to develop RCR training plans, but provides no guidelines or requirements for the format, scope, content, duration, or frequency of the training, and does not hold universities accountable for their training plans. Our study shows that this vaguely worded policy, and lack of accountability, has not produced meaningful educational experiences for most of the undergraduate students, graduate students, and post-doctoral trainees funded by the NSF.

**Electronic supplementary material:**

The online version of this article (doi:10.1007/s11948-017-9883-5) contains supplementary material, which is available to authorized users.

## Introduction

Since the middle of the 20th Century, the U.S. federal government has been an important worldwide funder of scientific research, particularly through the National Institutes of Health (NIH) and National Science Foundation (NSF). The U.S. government has also been an important force in promoting integrity and ethical conduct in research. Since the 1980s, Congress and individual agencies have issued a series of mandates that require individual research institutions to develop policies and programs to address research misconduct and promote research integrity (Heitman et al. 2015). The frequency of research scandals reported in academic forums, and the rising rate of publications retracted from scientific journals, highlights the continuing importance of policies and programs to promote integrity in every stage of a researcher’s career. It is equally important to evaluate these policies and programs. This project evaluates the NSF’s policy requiring institutions to provide responsible conduct of research (RCR) education to all NSF-funded trainees.

## Background

Institutional practices regarding RCR education vary considerably, but two federal agencies set minimum standards for institutions seeking federal funding for research projects. Since 1990, the NIH have required all applicants for National Research Service Award (NRSA) training grants to have a “program in the principles of scientific integrity” (NIH 1989, p. 1). More recently, the NSF has required universities to have an institutional plan to provide “appropriate training and oversight in the responsible and ethical conduct of research” to their NSF-funded trainees (NSF 2009b).

Despite their common educational goals, the two agencies developed their policies on RCR education separately and with significantly different levels of specificity. The NIH’s original 1989 policy on instruction in RCR called for institutions to provide instruction on topics relevant to research integrity through informal seminars and presentations or “formal courses on bioethics, research conduct, the ideals of science, etc.” (NIH 1989, p. 1). Five years later, the NIH updated this policy to require research training grant proposals to include much more detail about how they would provide instruction in RCR, including a description of the intended subject matter, format and frequency of instruction, expected participation of faculty and trainees, and the rationale for the chosen approach (NIH 1994). NIH also required competing and non-competing training grants to provide progress reports on the type of instruction provided, topics covered, and “other relevant information such as attendance by trainees and faculty participation” (NIH 1994).

The NIH updated and expanded its guidance again in 2009 to “convey some of the consensus best practices (in teaching RCR) that have evolved in the research training community over the past two decades” (NIH 2009). The revised guidelines, still in place today, provide even more specific standards on the format, scope, content, and duration, and frequency of instruction. They require a minimum of 8 h of face-to-face instruction on a comprehensive set of issues in research integrity, and specifically note that a plan that proposes online instruction alone is not acceptable (NIH 2009).

In contrast to the NIH, the NSF had few formal requirements for instruction in RCR before 2007. That year, Congress unexpectedly included a brief statement regarding RCR instruction in its provisions for funding NSF under the ‘‘America Creating Opportunities to Meaningfully Promote Excellence in Technology, Education, and Science Act’’ (better known as the ‘‘America COMPETES Act’’):

**SEC. 7009. RESPONSIBLE CONDUCT OF RESEARCH**

The Director shall require that each institution that applies for financial assistance from the Foundation for science and engineering research or education describe in its grant proposal a plan to provide appropriate training and oversight in the responsible and ethical conduct of research to undergraduate students, graduate students, and postdoctoral researchers participating in the proposed research project (America COMPETES Act of 2007).

In response to this congressional mandate, the NSF funded a workshop at the National Academy Engineering (NAE) in August 2008 to define the best practices for RCR instruction (Hollander and Bissell 2008). The workshop, entitled *Ethics Education: What’s Been Learned? What Should be Done?,* concluded that: (1) non-instructor-led, online-only programs do not provide adequate instruction; (2) multiple formats of instruction are needed; (3) programs should be wide-ranging and cross-institutional, with content that varies by disciplinary areas and career stage; (4) ethics education cannot be administered in a single “dose”; and (5) principle investigators (PIs) should be positively involved in teaching RCR to their trainees (Hollander et al. 2009; Feldman 2009).

The NSF published its proposed policy on RCR instruction in February 2009 (NSF 2009a) and, after a period for public comment, released its final policy in August 2009:

Effective January 4, 2010, NSF will require that, at the time of proposal submission to NSF, a proposing institution’s Authorized Organizational Representative certify that the institution has a plan to provide appropriate training and oversight in the responsible and ethical conduct of research to undergraduates, graduate students, and postdoctoral researchers who will be supported by NSF to conduct research. While training plans are not required to be included in proposals submitted to NSF, institutions are advised that they are subject to review upon request (NSF 2009b).

While the NSF engaged in significant outreach efforts to promote the best practices identified in the NAE’s workshop (Feldman 2009), its actual policy left the design of plans for RCR instruction entirely to the discretion of individual institutions. It provides no specific guidance or requirements for the structure, format, scope, content, duration, or frequency of required RCR training. Rather, as noted in a set of Frequently Asked Questions published in 2011, the broadly worded policy was based on the premise that “the research community… is best placed to determine the content of RCR training without a need for NSF-specified standards” (NSF 2011). Furthermore, rather than requiring institutions to describe the instructional plan in their proposals, the institution’s Authorized Organizational Representative (typically a staff member in an office of grants or sponsored programs) simply certifies that the university has a plan to provide oversight and training in “responsible and ethical conduct of research” for all NSF-funded trainees (NSF 2009, 2011). The PI is typically not involved in this certification process (NSF 2011).

Today, the scope and detail of NIH’s and NSF’s respective policies on instruction on the responsible conduct of research remain quite different. While there has been little comprehensive evaluation of RCR instruction nationwide (Mumford et al. 2015), national surveys have found that programs are quite variable and often lack coherency (DuBois et al. 2010; Resnick and Dinse 2012). Moreover, many institutions appear to have developed their RCR instruction with an eye toward a basic level of compliance with federal requirements (Resnick and Dinse 2012), rather than higher goals of excellence in science education (Bulger and Heitman 2007).

To determine the impact of the NSF’s policy on RCR instruction, this study analyzed the NSF-specific training plans in place at research-intensive universities in 2015, 5 years after the mandate of the America COMPETES Act’s took effect. Our objective was to determine how the academic research community responded to a governmental policy that intended to promote RCR training but left the details of the curriculum to the discretion of the academic institutions themselves. More specifically, we examined whether the instructional plans reflected the NAE’s consensus best practices regarding the structure, format, duration, and frequency of training in RCR.

## Method

Using the 108 institutions classified by the Carnegie Foundation in 2014 as a “research university with very high research activity (RU/VH)” (Carnegie Foundation 2014) as our primary sources of data, we collected the publicly-available NSF RCR training plans for these institutions through a comprehensive Internet search. Using the consensus best practices articulated at the National Academy of Engineering’s 2008 workshop as our analytic framework, we assessed each plan for its public accessibility, clarity, and structure; the format of the training the plan offered; the format of the training the plan required; whether the plan offered trainees choices about ways to fulfill the training requirement; whether the plan required the same training for all categories of trainees; and the frequency and duration of activities that satisfied its minimum training requirements. A more detailed version of the protocol is available in the supplemental materials (see Supplemental Materials_Protocol).

## Results

### Sample Characteristics

Of the 108 institutions classified as RU/VH by the Carnegie Foundation, 103 (95%) had plans for RCR instruction that were publicly available online during the period of April through September 2015. Ninety-three (93) of these could be found easily though links provided on the homepage of the institution’s research compliance office or by using the search function on the institution’s website; the remaining 10 were slightly more difficult to find within the institution’s website but could be located through a basic *Google* search using the university’s name and “RCR” as search terms. We were unable to find the NSF-required RCR training plans for 5 institutions: Rockefeller University, University of Arkansas, University of Illinois at Urbana-Champaign, University of Virginia, and Yeshiva University.

Of the 103 universities for which we located RCR training plans that addressed NSF’s policy, we found 5 universities (6%) did not provide institution-wide educational requirements. Four universities—North Dakota State University, Princeton University, University of California-San Diego, and University of Pennsylvania—had posted plans that did not state minimum requirements. For example, while the University of California at San Diego’s plan offered a wide array of educational resources and noted that each department and program determined its own requirements, the plan made no mention of what those requirements were. In contrast, Michigan State University’s (MSU) website detailed 16 discipline-specific plans, each with its own clearly stated minimum requirement, responding to the different needs of the university’s disciplines and programs. All 16 of the MSU plans were easily accessible on the university’s website, but the university did not describe a single “institutional” plan and thus is not included in our analysis.

We found an additional 7 universities (6%) had RCR training plans that were unclear: Northwestern University, University of Tennessee, University of Texas at Austin, University of Cincinnati, University of Louisville, University of Miami, and University of Oregon each had a plan that applied to all units in the institution, but which did not state its minimum requirements in a definitive way.

Thus, we analyzed the RCR instructional plans for 91 RU/VH institutions, which constituted 84% of the original cohort.

### Structure and Format of Institutional Plans

**Fig. 1:**
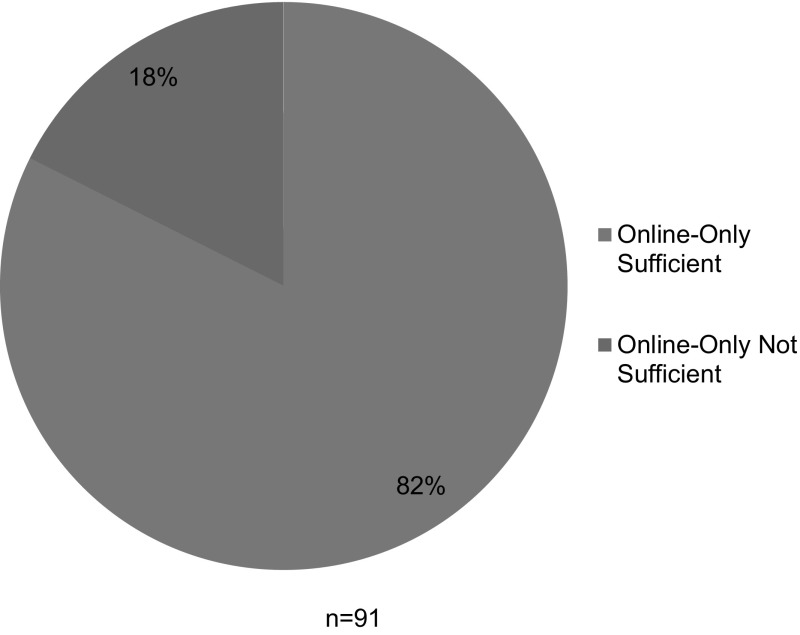
Is online-only training sufficient?

As shown in Fig. 1, 75 (82%) of the 91 RU/VH universities with available institutional plans for RCR instruction had plans that could be satisfied with online-only training. Among these 75 schools, 65 listed the Collaborative Institutional Training Initiative’s (CITI) RCR course as their only source of online instruction; the remaining 10 used an alternative online program, or a combination of online sources, or did not identify a specific source of online instruction.^1^

Many of the universities that required only online training used language to describe their requirement similar to that of Emory University’s plan:

All Emory University undergraduate, graduate students and postdoctoral researchers receiving NSF funds (salary/stipends) or NSF scholarship/stipend support to engage in research or if conducting research is included in their academic program that is receiving NSF support, are required to take the RCR training if the NSF grant proposal was submitted on or after January 4, 2010 are required to take the RCR training module available below.

*Those interested in fulfilling the NSF RCR training requirements can do so by completing the appropriate CITI RCR training course. Click here for instructions on how to take the CITI RCR course* (italics in original) (Emory 2016).

**Fig. 2:**
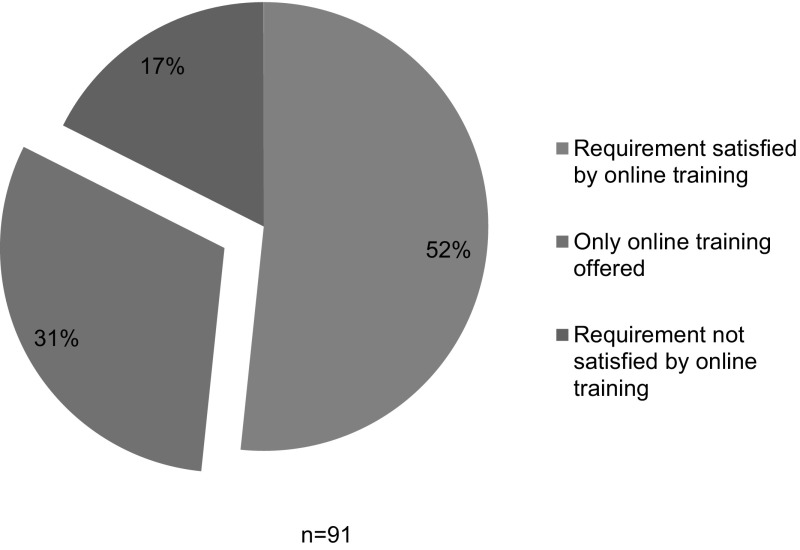
Differentiation among online requirements

This plan, and twenty-seven (27) others like it, listed the same requirements for undergraduate students, graduate students, and postdoctoral researchers, across all programs and units. They required only non-instructor-led online training provided by the CITI program’s RCR course, and offered no face-to-face training. Twenty-eight (28) of the plans that we analyzed, constituting 31% of the RU/HV universities with available institutional plans, used some variation of this language to describe their plans (see Fig. 2).

While nearly one-third of the institutional RCR training plans we analyzed offered nothing more than non-instructor led, online-only RCR training, two-thirds of the institutions were doing something more. A closer look at their RCR training plans reveals several interesting features regarding the structure and format of available educational opportunities in RCR.

### Uniform Versus Differentiated Plans

**Fig. 3:**
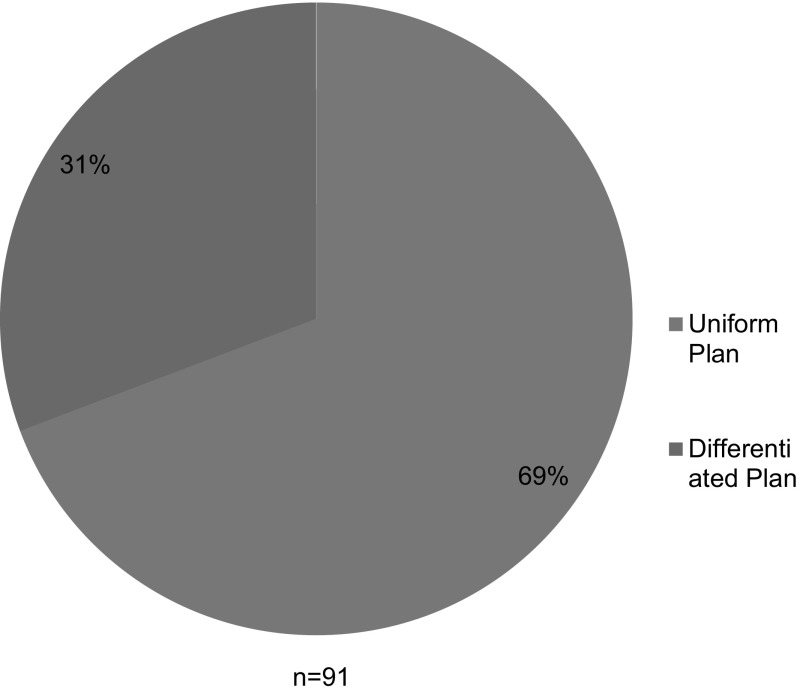
Uniform plan versus differentiated plan

Among the 91 institutions whose plans we analyzed, 63 (69%) had plans that set the same minimum RCR training requirements for undergraduate, graduate, and postdoctoral trainees; we described these as “uniform” plans. Twenty-eight (31%) institutions’ plans differentiated among levels of trainees, requiring different formats and duration of training for undergraduate students, graduate students, and postdoctoral fellows; we described these as “differentiated” plans (see Fig. 3). Three (3) of these 28 schools further differentiated between masters- and doctoral-level graduate students; the remaining 25 treated all graduate students as a single class of trainees.

**Fig. 4:**
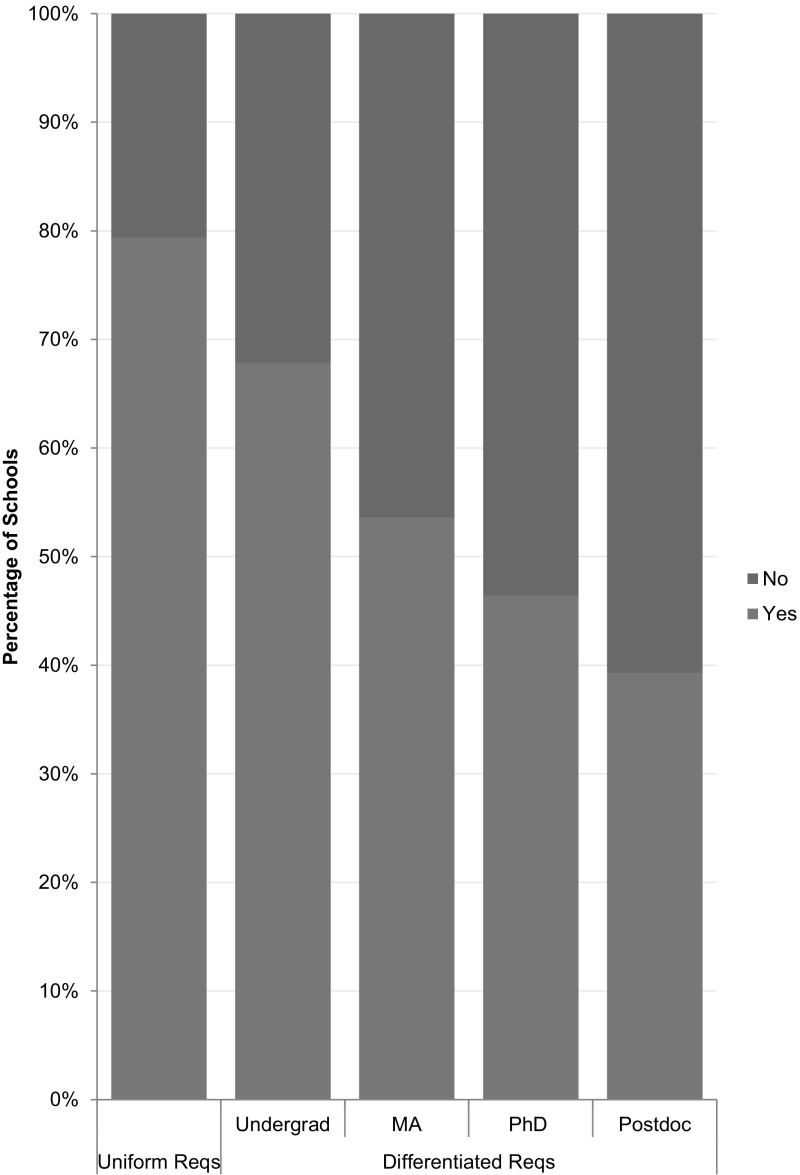
Is online only training sufficient?

Fifty (50) of the 63 schools with uniform plans (79%) required only online training, while 13 (21%) required some type of face-to-face instruction (see Fig. 4). Among the 28 schools that had differentiated requirements, 25 (89%) allowed online-only training for at least one class of trainees; but 19 (68%) required more than online only for at least one class of trainees (see Fig. 4). For example, Rice University had a uniform plan that required all classes of trainees to complete online-only training. Alternatively, Colorado State University had a differentiated plan that required undergraduate students to complete online-only training, but required graduate students and postdoctoral researchers to engage in a face-to-face supplement. Boston University required online-only training for undergraduate and masters-level graduate students, but required online instruction with a face-to-face supplement for doctoral trainees and postdoctoral researchers. While differentiated plans tended to require more instruction for the higher-level trainees, in at least one case we found the opposite: Pennsylvania State University’s plan considered online training for sufficient for postdoctoral researchers, but required additional instructor-led activities for other classes of trainees.

### Offerings Versus Requirements

In many of the plans that we analyzed we found a significant difference between the RCR training that institutions *offered* and the RCR training that they *required*. That is, many universities offered much more than they required.

**Fig. 5:**
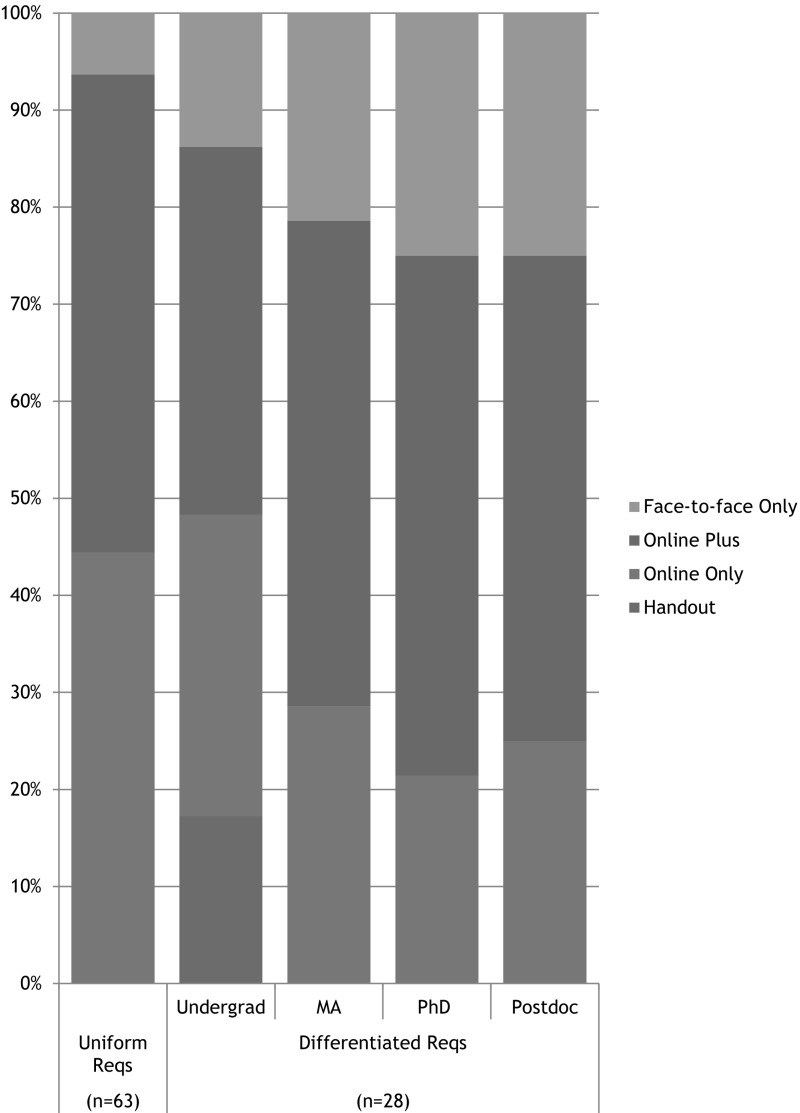
Educational offerings

Among the 63 universities with uniform requirements, 28 (46%) offered only online instruction; 31 (49%) offered online and face-to-face educational activities; and 4 (6%) offered only face-to-face activities (see Fig. 5). Among the 28 schools with differentiated plans across classes of trainees, 6 (21%) − 9 (32%) offered only online instruction for at least one group; 11 (39%) − 15 (54%) offered online and face-to-face activities, and 4 (14%) − 7(25%) offered only face-to-face activities (see Fig. 5). Notably, 22 (35%) of the uniform plans, and 13 (46%) of the differentiated plans *offered* face-to-face instruction *but did not require* such engagement (see Fig. 5). Alternative face-to-face offerings included seminar series; brown bag discussions; modules within an orientation program; dedicated sections of professional seminars; one-credit courses; three-credit courses; and extended orientations or retreats. At five (5) institutions, (University of California, Berkeley; University of California, Riverside; University of California, Santa Barbara, University of California, Santa Cruz, and University of Massachusetts Amherst), undergraduate students were able to meet the requirement for RCR instruction by receiving a hand-out, with no assessment of their comprehension of its contents. This “training” appeared less engaging than the CITI program’s online RCR course, which includes assessments for comprehension and retention.

### Structure of Plan: Single Path or Multi-Path

**Fig. 6:**
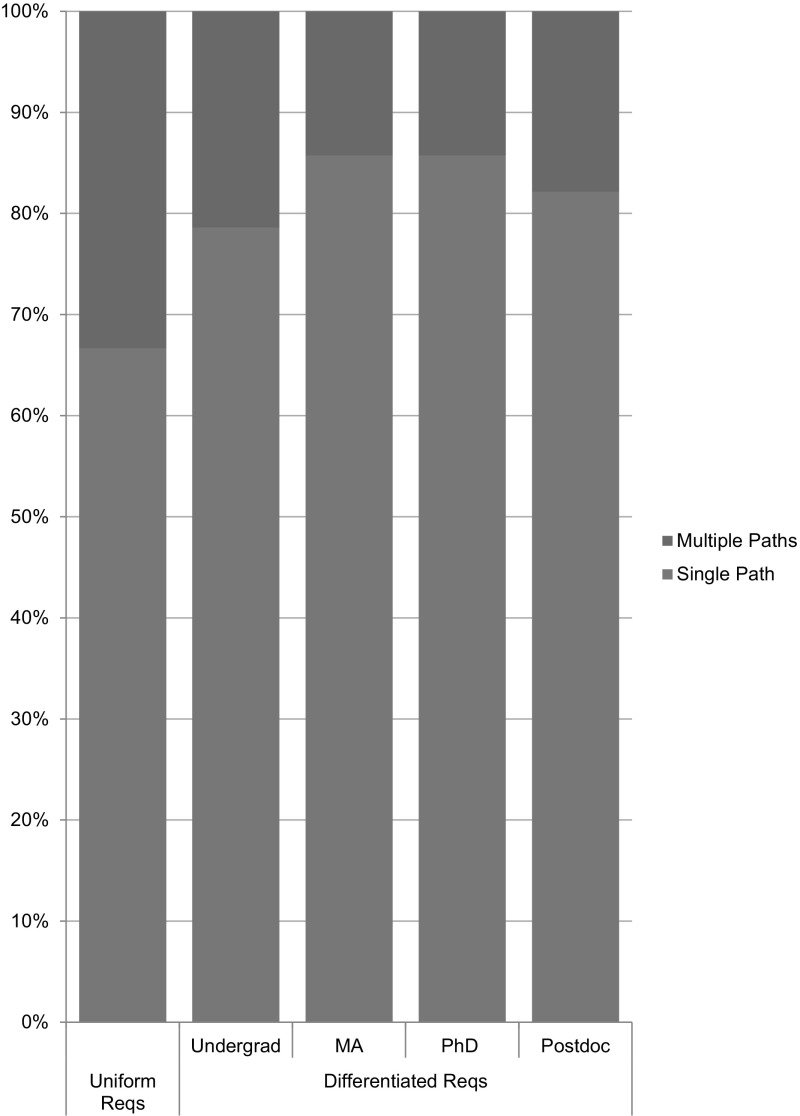
Single path versus Multi-path

As illustrated in Fig. 6, 66 (73%) of the plans that we analyzed presented a single path to fulfilling the institutional RCR training requirement. In 40 (44%) universities, this single path was online training, but at 5 (6%) universities, the only option was required face-to-face instruction (Johns Hopkins University, University of California-Davis, University of New Mexico, University of Oklahoma Norman Campus, and New York University). In twenty-seven (30%) of the 91 universities’ plans, students were offered multiple paths and could choose among more than one option to fulfill the training requirement. (Two schools in the differentiated group were included twice in this calculation because they offer a single path for some classes of trainees, and multiple paths for other classes of trainees.) This means that in some of the institutions for which online instruction is sufficient, students were offered the option to fulfill their training requirement in different ways. For example, Iowa State University's plan clearly stated that students have a choice: option 1 required successful completion of CITI’s online RCR course, while option 2 required completion of a one-credit (or greater) face-to-face course in RCR. Notably, not all of the universities that offered RCR instruction in multiple formats also offer multiple paths to satisfying the training requirement. For example, some institutions offered the CITI program’s online RCR course and face-to-face classes, but stated that all trainees must complete the CITI program. In these institutions, the face-to-face classes were supplemental, not alternatives to the required online training.

### Summary of Format and Structure of Plans

**Fig. 7:**
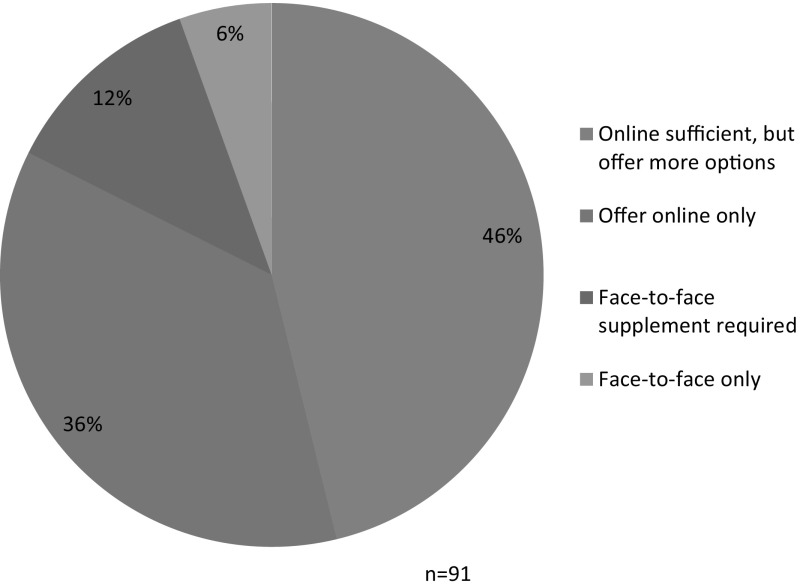
Overall picture

Among the 91 research intensive universities whose plans for RCR instruction we analyzed, 36 (40%) offered only and required only online training or printed handouts as fundamental instruction in RCR. This total includes the 31 universities with uniform plans; 1 university that required a different online resource for undergraduates (CMDITR) than it required for graduate students and postdoctoral researchers (CITI); and 4 universities that offered undergraduates a choice between receiving a printed handout or completing the online CITI course, but offered only the online CITI course to graduate students and postdoctoral researchers. Thirty-two (35%) universities either offered, but did not require face-to-face training, or required face-to-face instruction for some classes of trainees but not all. Eighteen universities (20%) required online training with face-to-face supplements for all trainees; and 5 (6%) offered and required only face-to-face instruction (see Fig. 7).

### Other Details of Institutional Plans

#### Duration

**Fig. 8:**
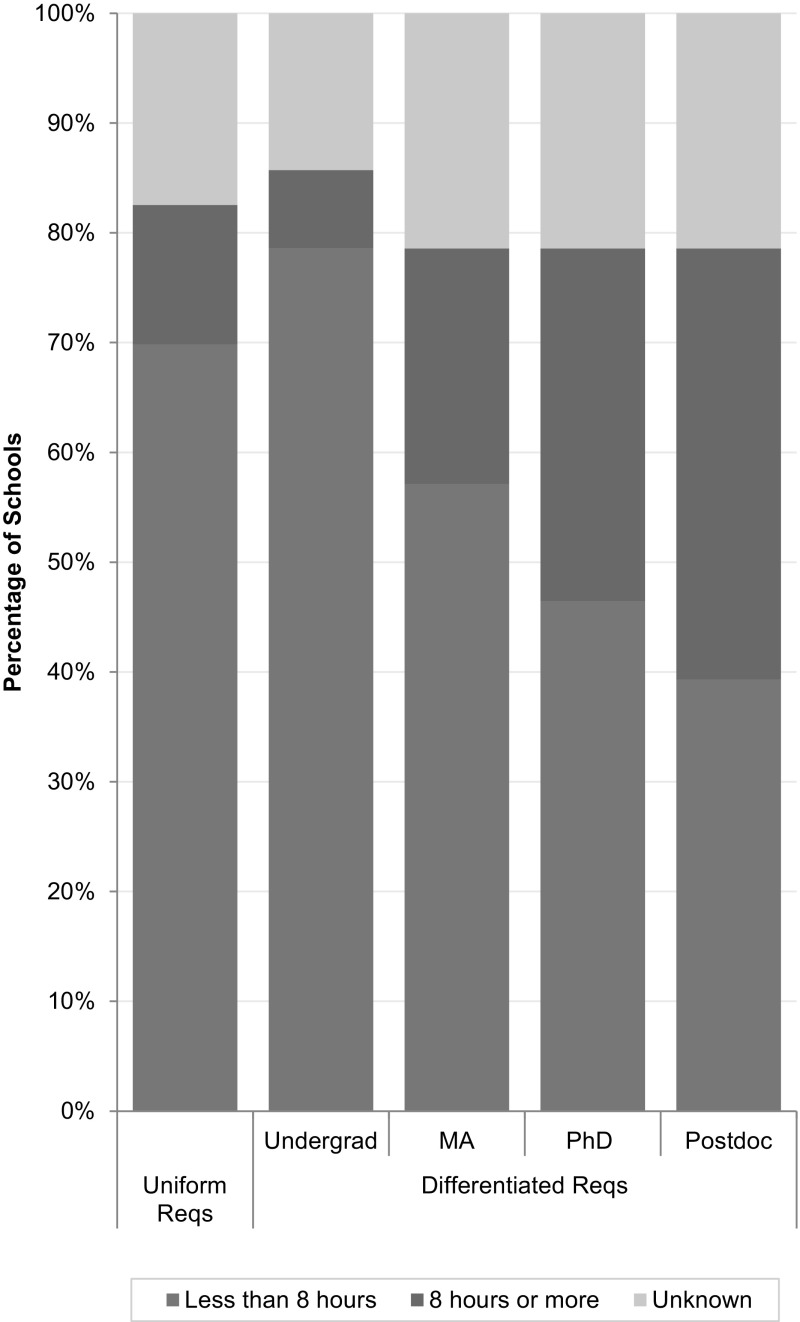
Duration of training

Nearly three quarters of all the institutional plans analyzed required less than 8 h of RCR instruction, as shown in Fig. 8. Among the 63 universities with uniform requirements, only 8 (13%) required 8 or more hours of training; among the 28 universities with differentiated requirements, only 11 (39%) required 8 h or more for at least one class of trainees.

#### Frequency

Among the 91 institutional plans we analyzed, 78 (86%) had requirements that could be completed with one-time-only training, while 13 universities (14%) required a renewal or refresher course for at least one class of trainees. Common intervals for refresher and renewal courses were 3, 4, or 5 years. Three universities required renewal or refresher activities at each career stage (University of North Carolina, Chapel Hill; University of Utah, and Virginia Commonwealth University); one institution (Washington University) required a renewal or refresher activity every year.

#### Source of Online Instruction

Among the 86 institutions that used online RCR training programs, we identified 75 (87%) that used the CITI program’s RCR course, 9 (10%) that used a different online resource,^2^ and 5 (6%) that referred to online training program in their plans but did not specify the source.

#### Time Frame

Among the 91 institutions’ plans for RCR instruction, 48 (53%) specified the time frame in which trainees must complete their instruction; 43 (47%) universities’ plans listed no time frame. The stated time frames range from “prior to employment” to “prior to end of award”; many were within 30 days, 60 days, or 1 year of the start of employment.

#### NIH Award Recipients

Of the 91 universities that had developed institutional RCR training plans in response to NSF funding requirements, 89 (98%) had also received an NIH training award in 2014. This indicates that there are sufficient RCR resources at these institutions for NIH trainees to develop RCR training programs that meet the NIH requirements regarding format, scope, content, and duration, and frequency of instruction (Table 1).

**Table 1:** Institutions for which online only training is sufficient

Sufficient for all trainees	Sufficient for at least one class of trainees	Insufficient for all classes of trainees
Brown University^a,b^ California Institute of Technology^a,b^ Carnegie Mellon University^a,b^ Case Western Reserve University^a,b^ Columbia University^a,b^ Cornell University^a,b^ Emory University^a,b^ Florida State University^a^ George Washington University^a,b^ Georgia State University^a,b^ Harvard University^a,b^ Iowa State University^a,b^ Louisiana State University Massachusetts Institute of Technology^a,b^ Montana State University^a,b^ North Carolina State University at Raleigh^a^ Ohio State University-Main Campus^a,b^ Rensselaer Polytechnic Institute^a,b^ Rice University^a,b^ Stanford University^a,b^ SUNY at Albany^a,b^ SUNY University at Buffalo^a,b^ Texas A & M University^a,b^ Tufts University^a,b^ Tulane University of Louisiana^a,b^ University of California-Irvine^a^ University of California-Los Angeles^a^ University of Central Florida^a,b^ University of Chicago^a,b^ University of Colorado at Boulder^a,b^ University of Delaware^a,b^ University of Florida^a,b^ University of Georgia^a,b^ University of Houston^a,b^ University of Illinois at Chicago^a,b^ University of Kansas^a,b^ University of Kentucky^a,b^ University of Maryland-College Park^a,b^ University of Massachusetts Amherst^a^ University of Michigan-Ann Arbor^a^ University of Minnesota-Twin Cities^a^ University of Missouri-Columbia^a,b^ University of Nebraska-Lincoln^a^ University of North Carolina at Chapel Hill^a,b^ University of Notre Dame^a,b^ University of Pittsburgh ^a^ University of South Carolina-Columbia^a,b^ University of South Florida-Tampa^a,b^ University of Southern California^a,b^ University of Washington^a^ University of Wisconsin-Madison^a^ Virginia Polytechnic Institute and State University^a,b^ Washington State University^a^ Wayne State University^a,b^	Arizona State University^a,b^ Boston University^a,b^ Brandeis University^a,b^ Colorado State University^a^ CUNY Graduate School^a,b^ Dartmouth College^a,b^ Duke University^a,b^ Georgetown University^a,b^ Pennsylvania State University ^a,b^ Purdue University-Main Campus^a,b^ University of Alabama at Birmingham ^a,b^ University of Alabama in Huntsville^a^ University of California-Berkeley^a,b^ University of California-Riverside^a,b^ University of California-Santa Barbara^a,b^ University of California-Santa Cruz^a,b^ University of Iowa^a,b^ University of Rochester^a,b^ Vanderbilt University^a,b^ Virginia Commonwealth University^a,b^ Yale University^a,b^	Georgia Institute of Technology-Main Campus^a,b^ Indiana University-Bloomington^a,b^ Johns Hopkins University^a,b,b^ Mississippi State University^a,b^ New York University^a^ Oregon State University^a,b^ Rutgers University-New Brunswick^a,b^ Stony Brook University^a,b^ University of Arizona^a,b^ University of California-Davis^a^ University of Connecticut^a,b^ University of Hawaii at Manoa^a,b^ University of New Mexico-Main Campus^a^ University of Oklahoma Norman Campus University of Utah^a^ Washington University in St Louis^a^
Total: 54	Total: 21	Total: 16

^a^Denotes 2014 recipient of NIH F, K, or T award

^b^Denotes use of CITI’s RCR course for online instruction

## Discussion

### Study Design

We collected the data presented here through Internet searches, rather than surveys or interviews with RIOs or institutional compliance staff, for two reasons. First, because many universities have put their course catalogues and essential institutional information on their websites, that that is where PIs and funded trainees are most likely to look for their requirements for RCR training. Administrators might communicate the relevant information about training plans to PIs and trainees in other ways, or RCR education requirements might be well-known features of departments and programs. However, this might not be the case at all institutions, so we were glad to find 84% of the plans clearly stated on institutional websites. An added advantage of publicly available institutional plans is that data collection for projects like this is much faster and less costly, with fewer missing observations when compared to typical survey response rates. We hope that the NSF will encourage, or require, institutions to post their training requirements on their websites to facilitate compliance and evaluative research.

Second, anecdotal reports indicate that PIs, trainees, compliance staff, and RIOs are confused about the difference between the educational activities that their universities offer generally and the training that their institutions require to meet NSF’s mandate under the AMERICA Competes. That is, many universities provide a wide variety of RCR-related educational opportunities, but these activities are not mentioned, offered, or required in their institutional plans for the NSF. Emory University is one such university: as an institution, it has an impressive portfolio of activities in RCR education, but the only activity offered and required under its NSF-mandated instructional plan is the CITI program’s RCR course. When asked about a university’s RCR plan in a survey or interview, a person might list all of the educational activities that the university offers, which might be different from what the university offers and requires in its NSF plan. For these institutions, interviews and surveys might have yielded different, and perhaps inaccurate, results about the specific impact of the NSF’s America COMPETES policy.

Our rationale for this design highlights an important concern about the impact of the NSF’s policy: in institutions that do not have strong cultures of research integrity, with RCR training requirements communicated and integrated into programs, PIs and trainees might not be aware of the NSF and institution’s policies. The NSF’s policy states that “grantees must have a plan in place to provide appropriate training and oversight in the responsible and ethical conduct of research,” and assurance that there is such a plan is typically provided by the office of sponsored programs, along with the many other federally-required assurances provided by the university—such as the university being a smoke-free workplace—as part of the overall submission process. In contrast, the NIH’s policy requires that the plan for instruction in RCR be described in a designated section of the proposal’s narrative and be scored as “acceptable” or “unacceptable” by reviewers. Thus a PI who submits a research training grant proposal to the NIH will be aware of both the NIH’s policy and the RCR training activities included in the plan. The NSF’s approach creates a risk that some, or perhaps many, PIs will not actively provide even minimal instruction in RCR because they do not know about the America COMPETES mandate, the NSF’s training requirement, or their institutional plan.

### Findings

The consensus best practices articulated by experts at the 2008 NAE/NSF workshop guided our analysis of the 91 institutional RCR training plans (Hollander and Bissell 2008; Hollander et al. 2009). These were accepted standards at the time the NSF’s policy was developed, as evidenced by not only by the workshop’s findings, but also by public comments to the proposed policy, the NSF’s own educational outreach, and the NIH’s update to its policy on RCR education later in 2009. Our findings indicate that the majority of research-intensive universities across the United States have implemented RCR training plans that fail to meet at least five of these best-practice criteria.

#### Non-instructor-led, Online-Only Programs do not Provide Adequate Instruction

The NAE workshop concluded that non-instructor-led, online-only programs “do not provide an adequate introduction or enough practical experience to prepare [trainees] for ethical problems that arise in academic and professional life” (Hollander et al. 2009, 38). This best practice is also reflected in the NIH’s 2009 policy update on instruction in the responsible conduct of research, which states that “online instruction is not considered adequate as the sole means of instruction” (NIH 2009). Despite this standard, 82% of the RCR training plans we examined could be satisfied by non-instructor led online training or even less interactive printed handouts (see Fig. 7).

#### Multiple Formats of Instruction are Needed

The report from the NAE’s 2008 workshop states that “institutions and researchers need a menu of programs, ranging from university-level to in-lab, informal, bench-level interactions, from which they can select the type of program most appropriate for their circumstances” (Hollander et al. 2009, p. 18). This best practice was also communicated in the NSF’s educational outreach (Feldman 2009). However, our findings show that many universities did not structure their plans to include multiple approaches to instruction in RCR. Only 28 of 91 universities (31%) offered at least one class of trainees a choice of how to satisfy the training requirement, although 49 (54%) institutions offered more than one type of training.

#### Programs Should be Wide-Ranging, Cross-Institution, with Content that Varies by Disciplinary Areas and Career Stage

The NAE’s workshop report and the NSF’s outreach materials state that the content of required RCR instruction should vary by discipline and career stage; this practice is also reflected in the NIH’s guidance, which states that RCR training plans should “optimize instruction in responsible conduct of research for the particular career stage(s) of the individual(s) involved” and be relevant to the research interest of the trainee (NIH 2009).

Of the 103 America COMPETES training plans that were publicly available at the time of our study, only one institutional plan, from Michigan State University (MSU), varied in content and requirements according to disciplinary area. In comparison to the other RU/VH institutions, this finding was so anomalous that we did not include MSU in the further analysis. Despite the NAE’s conclusion that content should be targeted to the trainees’ field of study, none of the other plans that we analyzed varied its content and requirements by discipline.

Of the 91 training plans that we examined, only 28 (31%) institutions had plans that varied the content and requirements according to the trainees’ career stage. Sixty-three (69%) had “one size fits all” plans, where a single plan covered trainees from all disciplines and all career stages.

#### Ethics Education Should not be Administered in a Single “Dose”

The report of the NAE’s 2008 workshop states that “ethics is not a vaccine that can be administered in one dose and have long-lasting effects no matter how often, or in what conditions, the subject is exposed to the disease agent” (Hollander et al. 2009, p. 36). Similarly, the NIH’s guidelines state that “Instruction (in RCR) must be undertaken at least once during each career stage, and at a frequency of no less than once every 4 years” (NIH 2009). Despite this recognized best practice, 78 (86%) of the institutions whose plans we analyzed employed a “single-dose inoculation” model.

#### PIs Should be Positively Involved in RCR Training Activities

While several of the plans encouraged PIs to be involved in their trainees’ instruction in RCR, none of the institutions required PI involvement. All of the training plans and related mandates were directed at trainees. PIs were not required to participate in training, conduct follow-up discussions with their trainees, or even be aware of the content of the RCR educational program.

In the end, we found that very few institutional training plans incorporated the best practices in RCR education that were generally known and promoted at the time that the NSF implemented its policy (Council of Graduate Schools 2006). It follows that very few institutional plans are likely to provide their trainees with a meaningful educational experience in the responsible conduct of research. Nearly half of the plans we reviewed *offered* more meaningful educational opportunities, but did not *require* that trainees engage in them. While these plans appear better than plans that did not offer additional activities, it cannot be assumed that PIs will encourage or require their trainees to seek additional RCR education beyond the institutional requirement, or that trainees will be self-motivated to engage in additional educational activities. Instead, the standards for meaningful education in RCR need to be reflected in the *minimum requirements* of the each institutional plan. When analyzed according to their minimum requirements, 82% of the RU/VH institutions we studied required nothing more for their trainees’ RCR instruction than the completion of a non-instructor-led online training module (see Fig. 1). Like the NSF policymakers, we want to believe that the research community is best placed to determine the appropriate content and structure of RCR training without federally defined standards. However, our data show that the academic research community is not implementing best practices in institutional training plans.

There are a number of reasons why institutions may not have incorporated recognized best practices into their RCR training plans. First, because the NSF’s mandate for RCR instruction appeared in the context of compliance standards, the architects of the plans may have lacked the necessary pedagogical expertise to develop strong training requirements in RCR, or may not have communicated with subject matter experts. Institutional compliance plans are often developed by administrators or staff members within an office of sponsored programs or institutional compliance; such officials may lack specialized knowledge about teaching and learning responsible research practices in science and the principles, concepts, and standards that have developed over the past two decades. Thus, the available consensus on best practices in teaching RCR may not have informed the development of many plans. In these cases, guidelines or more specific criteria from the NSF would have helped such institutions create plans that provide meaningful education in RCR.

A second possible explanation is the lack of a forum for sharing instructional experiences and resources. When faced with the mandate in 2009, university officials charged with developing an institutional plan for RCR instruction who wanted to know more about effective features and techniques may not have known where to turn for curricular resources and there was no quick way to find help. This concern was communicated in the public comments on NSF’s draft instructional policy in 2009, and the NSF responded by supporting the development of three online repositories of academic resources and curricular materials in RCR “to provide an interactive community location and searchable clearinghouse of resources on ethics education in science and engineering” (NSF 2009a, b). In 2009, NSF funded an online inter-disciplinary library of resources for teaching ethics at the University of Massachusetts Amherst (Fountain and Billings 2009). In 2009, the NSF funded the Online Ethics Center (OEC) at the National Academy of Science, Engineering, and Medicine (Hollander et al. 2009). And in 2010, NSF funded the National Professional and Research Ethics Portal, now known as the Collaborative Online Resource Environment or Ethics CORE at the University of Illinois Urbana-Champaign (Gunsalus et al. 2010).

Each of these repositories aimed to be a valuable resource for instructors. However, it is unclear whether administrators and other institutional personnel charged with developing, evaluating, and revising institutional plans find them useful. For example, while the OEC and Ethics CORE repositories are rich with resources for scholars and instructors of ethics in science, engineering, and research, they have few resources for administrators and architects of institutional plans. The limited selection of such materials is likely due to the paucity of research and scholarship on institutional best practices and program design as opposed to best practices in RCR education generally.

The director of the OEC has expressed an interest in collecting and sharing these resources once they are developed; but first they must be developed. This means that the research community needs more programs like the *Project for Scholarly Integrity* (CGS 2012a), as well as more funding opportunities to encourage and support this type of research. In the meantime, a forum for sharing plans and experiences would be a useful resource. For example, the principal investigator of this project (Phillips) was the co-architect (with Teresa Gammill) of an institutional plan that requires trainees either to complete the CITI program’s RCR modules and attend a series of face-to-face seminars, or take part in a face-to-face alternative in the form of a comprehensive course or departmental program. While this plan incorporates face-to-face instruction and multiple approaches, it never occurred to Phillips and Gammill to design a plan with requirements that varied by career stage; had they realized that this approach was possible, they would have incorporated the feature into the plan. Simply knowing what other institutions are doing, and how well their programs seem to be working, would help many universities in the development, evaluation, and revision of institutional plans.

A third possible explanation why institutional plans do not reflect best practices is financial resources. The NSF policy was a largely unfunded mandate, implemented at a time when many RU/HV universities were facing budget crises. Even if institutional administrators were familiar with best practices, and had an ideal plan in mind, they might have been unable or reluctant to dedicate financial resources to carrying it out. It is likely that most, if not all, of the institutions whose RCR training plans we analyzed already used the CITI program’s course on protection of human subjects in research for Institutional Review Board certification, and possibly for certification for their Institutional Animal Care and Use Committees, or good clinical practice or conflict of interest training. For these universities with current institutional subscriptions, the CITI RCR training modules were available at no additional cost. Thus, a CITI-only RCR program presented a cost-free compliance measure for the NSF policy.

Universities’ budgets are often tight, and allocations must be made with care, but it is not clear that a high-quality RCR plan will always require a significant financial commitment, especially for institutions that may already be engaged in relevant educational activities. Many of the universities in our sample had RCR activities at the departmental, college, and university levels that were not incorporated into their institutional plans. In these contexts, structuring a plan to include these activities would be unlikely to require a significant financial commitment. As part of the *Project for Scholarly Integrity*, the Council of Graduate Schools developed an “RCR Inventory,” which is an instrument that identifies the RCR education activities taking place at the departmental level, as well as users’ attitudes about the quality and sufficiency of these resources (CGS 2012b). This type of assessment would be a useful activity when developing or revising an institutional plan. Furthermore, some of the face-to-face instruction that we found in institutional plans requires little time and effort to develop and deliver. For example, a face-to-face seminar on the ethical standards of peer review could be delivered by a journal editor or board member from among the faculty, and is likely to be similar to something that she or he has already prepared. Better communication within institutions to identify existing resources would benefit the development of institutional plans.

A fourth possible explanation why institutional plans do not reflect best practices is that the NSF policy provides little accountability. According to the policy:

While training plans are not required to be included in proposals submitted to NSF, institutions are advised that they are subject to review upon request. (NSF 2009a, b)

The policy does not state criteria, or even expectations; and it does not establish a systematic or regular review process. Indeed, given the absence of stated criteria and expectations, a meaningful review process would be difficult because there would be no basis for determining a plan to be unacceptable. In this environment, there is little incentive for institutions to implement more robust requirements, especially when a CITI-only plan is easy and cost-free. Anecdotal accounts indicate that many administrators know that their plans do not incorporate best practices, and they intend to make improvements if and when the NSF requires them to do so; but otherwise, they view their plan as good enough for compliance.

If the NSF policy on RCR instruction included greater accountability, then institutions, principal investigators, and research training program directors might respond differently to the mandate. For example, if criteria and expectations were clearly stated in the Grant Proposal Guide (GPG), and if PIs were expected to develop a tailored plan to provide RCR education to the trainees supported by their project, and if these plans were reviewed as part of the overall proposal, then institutions and PIs might be more creative and engaged in the training, and the educational experience for trainees might be more meaningful.

## Conclusion

Federal policy has been an important driver of instruction in responsible conduct of research in U.S. universities for over 25 years and its impact on institutional efforts to promote research integrity has been significant. In keeping with “the best practices of the scientific community over the past two decades,” the National Institutes of Health’s training grant and career development programs have progressively raised the standards for the RCR instruction required in research training programs (NIH 2009). The NSF funded a review of “what’s been learned” in ethics education in science and engineering research and identified best practices that were similar to those of reflected in the NIH policy (Hollander and Bissell 2008, Hollander et al. 2009). Although the NSF’s subsequent policy called for its funded institutions to provide “appropriate training and oversight in the responsible and ethical conduct of research” to their NSF-funded trainees, the policy did not identify or require these best practices (NSF 2009a, b). Five years after the implementation, our analysis of RCR instructional plans for 91 top U.S. research universities shows that the majority of these universities have not implemented these best practices in their plans. It is time to rethink NSF’s policy with the original educational goals of the America COMPETES Act in mind.

## Electronic supplementary material

Below is the link to the electronic supplementary material.

Supplementary material 1 (DOCX 114 kb)

## Footnotes

1 The following institutions, whose RCR plans accept online training to satisfy their instructional requirement and offer no face-to-face resources, use an online alternative to the CITI program, as noted in parentheses: University of Michigan (PEERRS); University of Nebraska (GRDC 98); University of Pittsburgh (ISER); University of Wisconsin (Learn@UW); University of Washington (CMDITR, for undergrads only) Boston University (CITI + Blackboard quiz). From our initial analysis, it cannot be determined whether these online courses are instructor led, or how they compare to the more widely used CITI program…

2 Two institutions are counted twice in these figures because they use both the CITI program and another online resource. These institutions are Boston University, which offers a Blackboard program in addition to CITI, and University of Washington, which uses CMDITR for undergraduates and the CITI program for other classes of trainees.
