# Supplementary material for: America COMPETES at 5 years: An Analysis of Research-Intensive Universities’ RCR Training Plans
Source: Sci Eng Ethics. 2017 Mar 15;24(1):227–49. doi: 10.1007/s11948-017-9883-5 (PMC5799314; doi:10.1007/s11948-017-9883-5)
Supplement: Supplementary file 1 — Supplementary material 1 (DOCX 114 kb) [file 11948_2017_9883_MOESM1_ESM.docx]

**Protocol**

Article Title: America COMPETES at 5 Years: An Analysis of Research-Intensive Universities’ RCR Training Plans

Data Collection

Our sample began with all 108 institutions classified as “research university with very high research activity” (RU/VH) by the Carnegie Foundation.

Using publicly available information and basic Internet searches, we searched for the NSF RCR training plans for these institutions. We started with institutional home pages and looked for links to research integrity or research compliance office pages. On these sites, we looked for mentions of responsible conduct of research training and the institutional response to the America Competes Act. If we found nothing referencing required training using this approach, we used institutional website search engines to look for “America Competes Act,” “National Science Foundation,” “responsible conduct of research,” “RCR training,” and “RCR NSF.” Finally, if this second approach yielded nothing, we moved from institutional websites to Google, searching for the institution name plus each of the search terms listed above. This method yielded NSF RCR training plans for 103 institutions, which we saved as screenshots or PDF files. We were unable to find NSF RCR training requirements for the remaining 5 institutions.

In order for researchers and trainees to comply with NSF grantee requirements, they must first be able to find their institutions’ training RCR training plan. Therefore, as we searched for the training plans, we took note of how easy they were to find. We coded the public accessibility of the plan according to the following criteria:

| *Easy:* | America Competes Act response located on first webpage readers are taken to after clicking “research” on the school homepage and then “training” or “compliance” or “integrity” on the next webpage, if these links (or links using similar language) are available, or |
| --- | --- |
|  | America Competes Act response can be found on first page of results when using school search engine and searching for “America Competes Act” or “RCR training” or “RCR NSF” or “responsible conduct of research” |
| *Medium:* | Institution’s response to America Competes Act can be found on first page of results by using Google and searching for school name plus search terms listed above |
| *Difficult:* | Institution’s response to America Competes Act cannot be found using the above approaches |

We were unable to find institutional plans classified as “difficult” and therefore did not include in our coding sample.

Data Coding and Analysis

Using data gathered and saved as screenshots or PDFs, we coded plans according to the following categories.

*University Wide Plan*

Some institutions do not have a university-wide training plan for America COMPETES compliance. Instead, these institutions allow individual departments and colleges to create their own training plans, with no university wide minimum requirements. We coded these plans as “no university wide plan” and because the offerings and required minimums are not university wide, we did not continue to code the plans for these institutions.

*Clearly Stated Requirements*

There were some institutional plans that were both publicly available and university-wide, but so unclear in their language that we could not identify minimum requirements. Once we determined that a plan was unclear we did not continue to code the plans for these institutions.

*Uniform Plan or Differentiated Plan*

Some institutions have plans that set the same minimum RCR training requirements for undergraduate, masters and doctoral graduate students, and postdoctoral researchers. These were coded as “uniform” plans. Other institutions differentiate classes of trainees, offering and requiring different formats and minimum levels of training for undergraduates, graduate students, and postdocs. Plans of this type were coded as “differentiated” plans.

*Single Path or Multi-Path*

This category captures whether institutional plans offer the NSF trainee a choice among training formats when fulfilling minimum requirements. For example, some institutions allow the trainee to choose between online or face-to-face options for RCR training. These institutions were coded as “multi-path.” Alternatively, some institutions offer only one option for fulfilling minimum requirements. Institutional plans that allow trainees no choice were coded as “single path.”

Single path plans can be of any format; the key is whether trainees have a choice of format. Institutions coded as single path might require online-only training, or face-to-face training, or a combination—they are single path not because of the format they require but because trainees have no training alternative to the stated format. Institutional plans that provide trainees choice in training format; that is, multiple paths that trainees can choose from to fulfill minimum training requirements, are “multi-path.” In these cases, trainees can opt for either online or face-to-face training and be compliant.

*RCR Education Offerings*

Some institutions may provide additional RCR education offerings beyond their required format. This category captures the fact that for many plans *requirements* and *offerings* are not necessarily the same. For example, an institution might require all NSF trainees to take online training, but might offer additional courses on RCR education as supplements to that training. We used the following coding scheme to note all formats offered for RCR education and noted in plans:

| O | Online training is offered |
| --- | --- |
| F2F_S | Face-to-face training is offered as a supplement (not alternative) to required online training. These training opportunities include seminars, discussion forums, brown bag sessions, and for-credit or not-for-credit courses. |
| F2F_A | Face-to-face training is offered as an alternative to (or in the absence of) online training. These training opportunities typically include for-credit or not-for-credit courses. |
| HO | RCR educational handouts are offered. |
| OTH | “Other”: RCR education is offered in another format not captured through the above coding scheme (such as PowerPoint presentations with no assessment.) |

Importantly, we noted face-to-face supplement or face-to-face alternative options only where specific courses or trainings were mentioned or listed. Vague references to “encouraged discussion,” “encouraged course development,” or “planned course development” were not counted as offerings.

*Is Online-Only Training Sufficient?*

Given that best practices indicate online-only training is not sufficient for effective RCR education, this category captures whether institutions whether online-only training meets an institution’s minimum requirements. For uniform plans, we noted our findings with a simple “yes” or “no”; we also noted the cases where less than online-only training meets the minimum standard (in some cases merely obtaining a handout is sufficient). For differentiated plans, sometimes online-only training is all that is required for some classes but not for others. For these plans, we noted whether online-only training is sufficient for each class of trainee.

*Source of Online Training*

This category captures whether institutions offering online training are using the Collaborative Institutional Training Initiative or another resources. These were notes as CITI or Other. Some institutions do not mention online offerings, and in these cases the source of online training was noted as “none.”

*Frequency*

Best practices suggest RCR training is better if provided more than once. We noted whether institutions require renewal and refresher training, or whether completing RCR education once is sufficient.

*Duration*

NIH requires that trainees complete eight or more RCR training hours, however, the NSF does not stipulate a minimum training duration. This category captures whether institutional plans required less than eight hours of training. Because the CITI program offers 4 continuing medical education (CME) credits for the completion of the RCR online training module, we counted the CITI program as four hours of training. Therefore, institutions that require only CITI training as their minimum were coded “less than eight.” Institutions that require 4 hours of face-to-face training beyond CITI were thus coded as “8 or more.” Institutions for whom the source of online training was “other,” and institutions for whom we could not determine the duration of the required supplements or alternatives were coded as unknown.

*Time Frame*

This category captures whether the training plan clearly states a deadline by which trainees must to complete their training. This information is both useful to trainees and supervising researchers, and ensures that training is completed in a timely manner. For example, plans stating that trainees must complete their training before starting work, within a certain number of days or months from starting, or before beginning their third semester of coursework were coded as “stated in plan.” Plans that did not mention any kind of timeline were coded as “not stated in plan.”

*NIH Award Recipients*

Institution that receive NIH training grants obviously have the resources to offer more robust RCR training, because the applications require a training plan that includes at least 8 hours of training, on a variety of topics, and not entirely online. Because of these stringent NIH requirements, we thought that institutions with NIH training awards would demonstrate an unwillingness to use online-only training for their NSF trainees. To test this idea, we generated a list of training award recipients and cross-referenced the list with the 91 Carnegie RU/VH institutions that we coded.
